# LASSO-based screening for potential prognostic biomarkers associated with glioblastoma

**DOI:** 10.3389/fonc.2022.1057383

**Published:** 2023-01-16

**Authors:** Yin Tian, Li’e Chen, Yun Jiang

**Affiliations:** ^1^ Department of Pediatric Surgery, Jingzhou Central Hospital, Jingzhou Hospital Affiliated to Yangtze University, Jingzhou, Hubei Province, China; ^2^ Department of Pathology, Sanya Central Hospital (Hainan Third People‘s Hospital), Sanya, Hainan Province, China; ^3^ Department of Ultrasound Diagnosis, Hubei Provincial Hospital of Integrated Chinese & Western Medicine, Wuhan, Hubei Province, China

**Keywords:** GBM, LASSO, independent prognostic factors, RPL39L, NUDT5

## Abstract

**Background:**

Glioblastoma is the most common malignancy of the neuroepithelium, yet existing research on this tumor is limited. LASSO is an algorithm of selected feature coefficients by which genes associated with glioblastoma prognosis can be obtained.

**Methods:**

Glioblastoma-related data were selected from the Cancer Genome Atlas (TCGA) database, and information was obtained for 158 samples, including 153 cancer samples and five samples of paracancerous tissue. In addition, 2,642 normal samples were selected from the Genotype-Tissue Expression (GTEx) database. Whole-gene bulk survival analysis and differential expression analysis were performed on glioblastoma genes, and their intersections were taken. Finally, we determined which genes are associated with glioma prognosis. The STRING database was used to analyze the interaction network between genes, and the MCODE plugin under Cytoscape was used to identify the highest-scoring clusters. LASSO prognostic analysis was performed to identify the key genes. Gene expression validation allowed us to obtain genes with significant expression differences in glioblastoma cancer samples and paracancer samples, and glioblastoma independent prognostic factors could be derived by univariate and multivariate Cox analyses. GO functional enrichment analysis was performed, and the expression of the screened genes was detected using qRT-PCR.

**Results:**

Whole-gene bulk survival analysis of glioblastoma genes yielded 607 genes associated with glioblastoma prognosis, differential expression analysis yielded 8,801 genes, and the intersection of prognostic genes with differentially expressed genes (DEG) yielded 323 intersecting genes. PPI analysis of the intersecting genes revealed that the genes were significantly enriched in functions such as the formation of a pool of free 40S subunits and placenta development, and the highest-scoring clusters were obtained using the MCODE plug-in. Eight genes associated with glioblastoma prognosis were identified based on LASSO analysis: RPS10, RPS11, RPS19, RSL24D1, RPL39L, EIF3E, NUDT5, and RPF1. All eight genes were found to be highly expressed in the tumor by gene expression verification, and univariate and multivariate Cox analyses were performed on these eight genes to identify RPL39L and NUDT5 as two independent prognostic factors associated with glioblastoma. Both RPL39L and NUDT5 were highly expressed in glioblastoma cells.

**Conclusion:**

Two independent prognostic factors in glioblastoma, RPL39L and NUDT5, were identified.

## Introduction

Glioblastoma is a malignant primary brain tumor disease and is among the most common malignant tumors of the central nervous system (CNS) (1). It accounts for 30% of all brain and CNS tumors and 80% of all malignant tumors (2). Despite the current multimodal treatment modalities, patients with glioblastoma have a poor prognosis, with a median survival time of only 14.6 months (3). The median age at diagnosis is 64 years (4), and the five-year mortality rate is higher than 90% (5). It has been shown that the incidence of glioblastoma is higher in men than in women (4, 6), the incidence is higher in developed countries than in developing countries (4), and the incidence of glioblastoma is higher in Asians, Latinos, and whites (7). Glioblastomas are aggressive tumors with a median survival time of only three months if left untreated (8). Surgery, radiation, and chemotherapy are available to improve the survival rates of glioblastoma patients.

The clinical treatment of glioblastoma may be facilitated by identifying genes and independent factors associated with glioblastoma prognosis. In this study, glioblastoma related data were selected from the Cancer Genome Atlas (TCGA) database, which contains 153 tumor samples and 2,647 paraneoplastic and GTEx undiseased tissues. Whole-gene bulk survival analysis of glioblastoma genes was performed to identify genes associated with glioblastoma prognosis. Differential expression analysis of the genes was conducted after whole-gene bulk survival analysis was performed with genes from normal samples. The interaction network between genes was analyzed using Metascape, and the highest- scoring clusters were identified using the MCODE plug-in. GO functional enrichment analysis was performed, and LASSO prognostic analysis was performed to identify key genes. Gene expression validation was performed to identify genes with significant expression differences in glioblastoma cancer samples compared with paracancerous samples, from which independent prognostic factors associated with glioblastoma prognosis were identified. Finally, qRT-PCR was used to verify the expression of the genes.

Due to the heterogeneity and complex pathogenesis of glioblastoma, the disease is still incurable (9). In this study, we explore the prognosis-related genes and independent prognostic factors of glioblastoma based on the LASSO bioinformatics analysis method.

## Methods and materials

### Sample source

This study is an exploratory study based on TCGA (https://portal.gdc.cancer.gov/) dataset, which contains 153 tumor samples, 5 paracancerous tissues, and 2647 normal samples of undiseased tissues retrieved from Genotype-Tissue Expression(GTEx, https://commonfund.nih.gov/GTEx) undiseased tissues.The 153 tumor samples were analyzed along with five samples of paracancerous tissues after obtaining gene expression matrices and clinical information data to identify genes associated with glioblastoma prognosis.

### Glioblastoma whole-gene bulk survival analysis

RNA-seq data of glioblastoma and corresponding clinical sample information were obtained from the TCGA, and the whole-gene bulk survival of the dataset was analyzed using the R package ggplot.2 Images were plotted using the R package forestplot. The data obtained from the all-gene bulk survival analysis could be used for subsequent analyses. A value of p<0.05 was considered statistically significant.

### Glioblastoma differential expression gene analysis

Clinical information was used to classify the samples into disease and control groups, and the Limma software package for R computing was used to analyze the differential expression of the study mRNAs. The results of the differential expression analysis for each data set are presented by volcano plots, and a Venn diagram shows the overlapping portion of differentially expressed genes (DEG) in the two groups; the overlapping genes can be used for subsequent analysis. The screening threshold for DEG is P<0.05, |log2FC|>1.

### Glioblastoma protein–protein interaction network analysis

To further investigate the genes associated with glioblastoma functional pathways, we performed PPI analysis on overlapping genes. Overlapping genes were analyzed using STRING (https://cn.string-db.org/) to obtain a protein-protein interaction relationship network. The relationship network was imported into Cytoscape for visualization, and the densely connected network components were identified using the MCODE plug-in to obtain seven gene modules, and the highest-scoring gene module was selected for subsequent analysis. Since the relationship network map drawn by STRING was more complex, the protein-protein interaction network map was redrawn using Metascape (https://metascape.org/).

### Glioblastoma prognostic risk model construction

LASSO (least absolute shrinkage and selection operator) is a risk-scoring model based on prognostic factors (10). The analysis used the R software survival package to conduct a multivariate Cox regression analysis, followed by an iterative analysis using the STEP function, thus selecting the optimal model as the final model. The prognostic risk model was constructed using the highest-scoring gene module obtained by the PPI algorithm to obtain a risk scoring formula. For Kaplan–Meier curves, p-values and hazard ratios (HR) with 95% confidence intervals (CI) were derived by the log-rank test and univariate cox regression. A value of p<0.05 was considered statistically significant.

### Comparison of glioblastoma gene expression

A comparative expression analysis of genes in the LASSO formula was implemented using the R software ggplot2 package to compare the distribution of the same gene in tumor tissue and normal tissue. A value of p<0.05 was considered statistically significant.

### Finding independent prognostic factors for glioblastoma

To further investigate the independent prognostic factors of glioblastoma, univariate Cox and multivariate cox regression analyses of genes were performed using the R software forestplot package; the results are presented using forest plots. A factor was considered to be an independent prognostic factor when the P value for both univariate cox and multivariate cox was less than 0.05.

### Cell culture

Human microglia HMC3 cells (Procell, CL-0620) were used as the experimental group (Experimental), and human U251 glioblastoma cells (Procell, CL-0237) comprised the control group (Control). HMC3 cells were cultured in high-glucose Dulbecco’s modified Eagle medium (DMEM; Hyclone, Shanghai, CHN) and supplemented with 1% penicillin–streptomycin (Solarbio Life Sciences, Beijing, CHN) and 15% fetal bovine serum (FBS; GIBCO, Grand Island, USA). U251 cells were cultured in penicillin (100 μg/ml) and streptomycin (100 μg/ml) in high-sugar DMEM and 10% FBS. All cell lines were grown at 37°C in 5% CO2.

### Detection of RPL39L and NUDT5 gene expression by qRT-PCR

Total RNA was extracted from both groups of cells using TRIzol reagent (Invitrogen, USA). The extracted mRNA was reverse transcribed into cDNA using SuperReal PreMix Plus (SYBR Green) (FP205-02, Tiangen, China), and gene expression was detected using qRT-PCR. The relative expression of the genes was calculated using the 2^-ΔΔCT^ method. The experiment was repeated three times to determine the average. The expression levels of RPL39L and NUDT5 were detected using GAPDH as an internal reference. The primer sequences used are shown in **Table 1**.

**Table 1:** Primer sequences of genes.

Gene	Forward	Reverse
RPL39L	5’-CAAAATCGTCCCATCCCC-3’	5’-TTCTTCTCCAATGCCTCCTT-3’
NUDT5	5’-GTTCTCCAGCGGTCTGTATG-3’	5’-CTTCGGCCTTGCGTTTTCG-3’
GAPDH	5’-CCTCTCCAGAACATCATCC-3’	5’-GTGTCGCTGTTGAAGTCAG-3’

## Results

### Glioblastoma whole-gene bulk survival analysis and differentially expressed gene analysis

**Figure 1:**
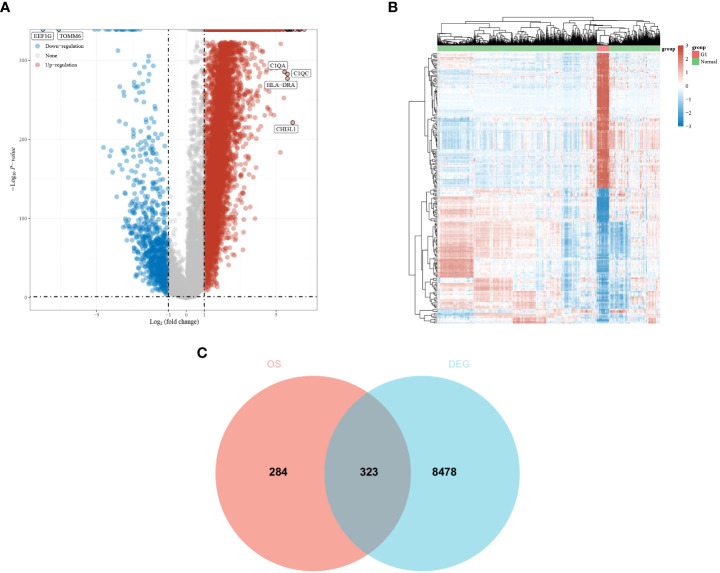
Differential expression analysis of glioma dataset to obtain DEGs. **(A, B)** Differential expression analysis volcano and heat map. The volcano plot in red indicates up-regulated genes, and that in blue indicates down-regulated genes. **(C)** An intersection of prognostic genes obtained from whole-gene batch survival with DEGs was taken. Red indicates prognostic genes, and blue indicates DEGs.

A total of 607 genes significantly associated with prognosis were obtained from a whole-gene bulk survival analysis of 153 glioblastoma tumor samples. To further analyze the distribution of genes in glioblastoma compared with their distribution in paraneoplastic and normal tissues, a differential analysis was performed. The results showed that there were 7,919 upregulated genes and 882 downregulated genes (**Figure 1A**). The differential expression heat map demonstrated the expression trends of the largest 50 upregulated and 50 downregulated genes in different tissues (**Figure 1B**). Three hundred and twenty-three genes that overlapped two groups were identified using a Venn diagram (**Figure 1C**).

### Results of PPI analysis

**Figure 2:**
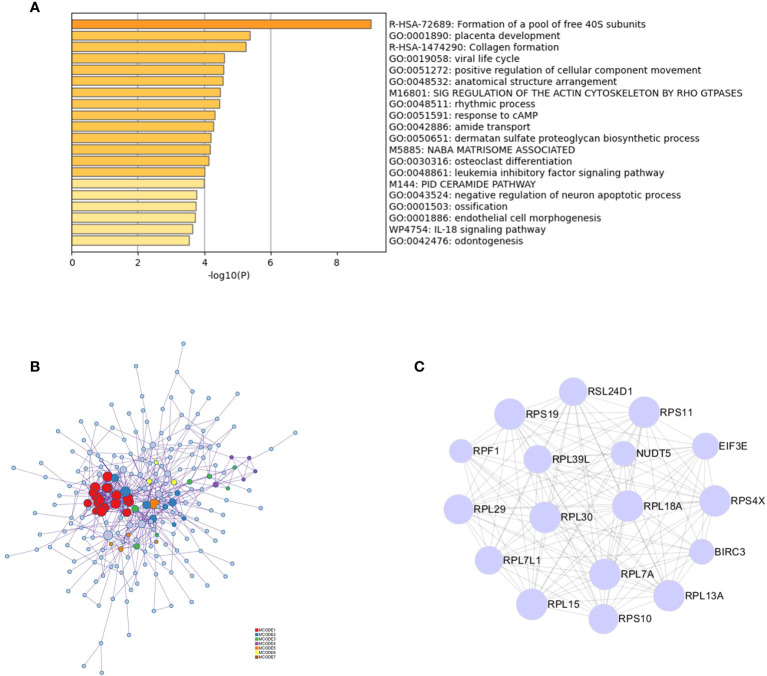
PPI analysis of intersecting genes. **(A)** Functional and pathway enrichment analysis of 323 genes using Metascape. **(B)** Analysis of the interactions between 323 genes using Metascape. Modules based on the MCODE algorithm are shown in different colors. **(C)** Analysis based on the MCODE algorithm in Cytoscape to obtain the highest scoring gene modules.

Functional enrichment analysis of 323 DEGs using Metascape revealed that the genes were significantly enriched in functions such as the formation of a pool of free 40S subunits and placenta development (**Figure 2A**). Protein–protein interaction expression was constructed for 323 genes to identify a gene-to-gene interaction linkage, as illustrated in **Figure 2B**. Nineteen gene modules were obtained *via* the MCODE algorithm, and the highest-scoring gene module was selected, which contained 17 nodes and 124 edges (**Figure 2C**).

### LASSO analysis to identify genes associated with prognosis

**Figure 3:**
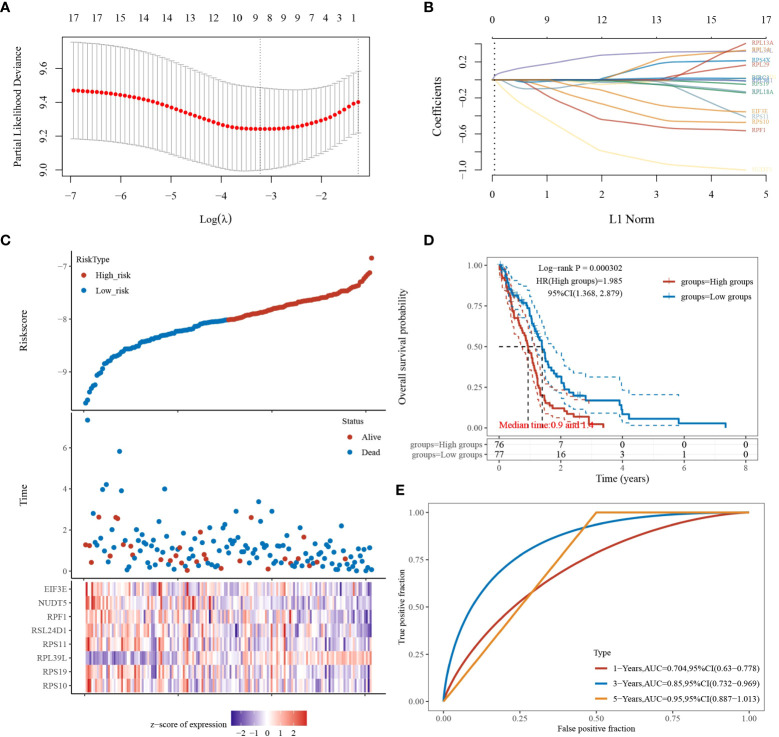
LASSO analysis of the 17 genes derived from PPI analysis. **(A)** The coefficients of selected features are shown by the lambda parameter, the horizontal coordinate represents the value of the independent variable lambda, and the vertical coordinate represents the coefficient of the independent variable. **(B)** The partial likelihood deviation was plotted against log(λ) using the LASSO Cox regression model. **(C)** Risk score, survival time, and survival status in the selected dataset, presented using scatter plots and heat maps. **(D)** KM survival curves with median risk values as groupings. **(E)** ROC curves for the risk model.

Based on the above study, 17 genes associated with glioblastoma were identified, and a prognostic model was constructed using multivariate cox regression analysis. The coefficients of the selected features were shown by the λ parameter; partial likelihood deviations were plotted against log(λ) using the LASSO Cox regression model (**Figures 3A, B**). The risk score formula is as follows: Riskscore = (-0.2159)*RPS10 + (-0.0121)*RPS19 + (0.2469)*RPL39L + (-0.0443)*RPS11 + (-0.0357)*RSL24D1 + (-0.3645)*RPF1 + (-0.6918)*NUDT5 + (-0.0858)*EIF3E. Based on the calculation of the risk score formula, the sample was divided into high-risk and low-risk groups; the distribution of the sample is illustrated in **Figure 3C**. **Figure 3D** demonstrates that the low-risk group is effective for prognosis. **Figure 3E** demonstrates that the model is accurate in predicting the three- and five-year survival of glioblastomas.

### Independent prognostic factors for glioblastoma

**Figure 4:**
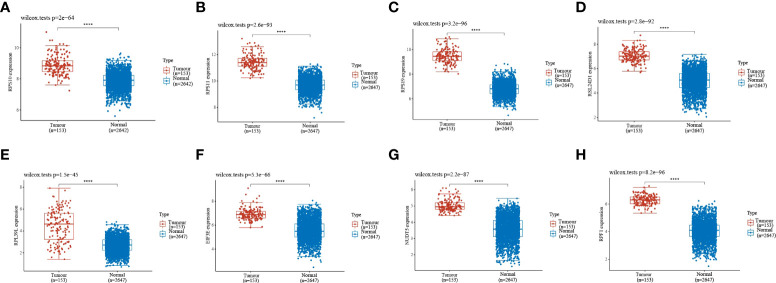
Expression validation of the eight genes in the LASSO formula. **(A–H)** The expression validation of the eight genes in the LASSO formula showed that all eight genes were highly expressed in gliomas. Red indicates tumor samples, and blue indicates normal samples. Prognostic gene expression validation. ****indicates P<0.0001.

**Figure 5:**
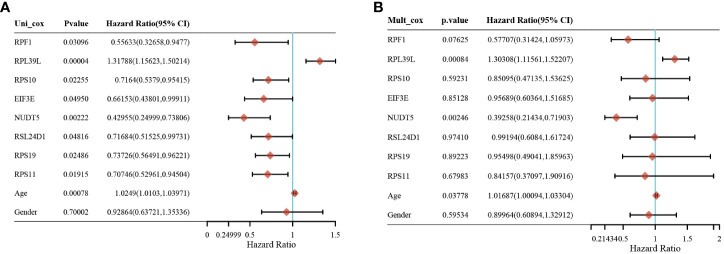
Screening of independent prognostic factors for glioma based on univariate and multivariate cox analyses. **(A)** Univariate cox analysis of prognostic genes. **(B)** Multivariate cox analysis of prognostic genes.

Gene expression analysis of eight genes obtained from LASSO that were associated with glioblastoma prognosis was performed, and all eight genes were found to be highly expressed in the tumors **(Figures 4A–H)**. These genes are presented as box line plots. An independent prognostic analysis of these eight genes was performed to obtain the results of univariate **Figure 5A** and multivariate Cox **Figure 5B** analyses, which are presented as forest plots. As can be seen in the figure, two genes, RPL39L and NUDT5, were significant in both univariate and multivariate Cox regression; therefore, these two genes can be considered independent prognostic factors of glioblastoma.

### Results of qRT-PCR

**Figure 6:**
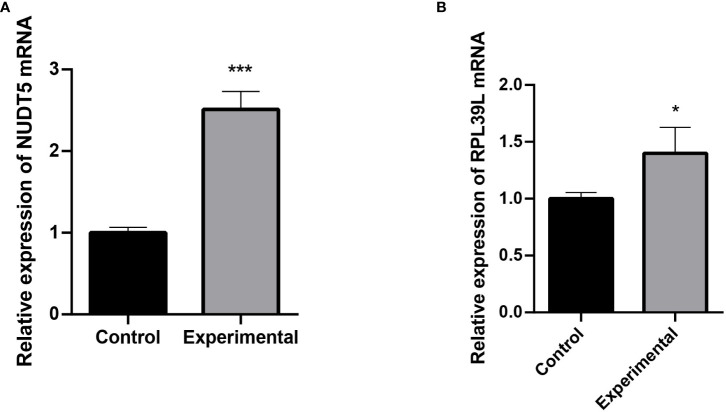
Detection of RPL39L and NUDT5 expression using qRT-PCR. **(A)** Relative expression of NUDT5 mRNA. **(B)** Relative expression of RPL39L mRNA. *** indicates P<0.001 compared with the control group; * indicates P<0.05 compared with the control group.

The expression of RPL39L and NUDT5 in glioblastoma cells and normal cells was detected using qRT-PCR. The results showed that both genes were highly expressed in glioblastoma cells (**Figures 6A, B**).

## Discussion

In recent years, the number of cancer patients has increased dramatically, and finding a breakthrough treatment for cancer has become urgent (11). Malignant glioblastoma is the most common type of primary brain tumor in adults and is associated with a disproportionate amount of cancer-related morbidity and mortality (12), making it particularly important to find ways to treat glioblastoma tumors. The rising trend in the incidence of glioblastoma has been accompanied by an increase in concern (2). At present, the diagnosis of glioblastoma still depends mainly on pathological features and medical imaging, such as CT, MRI, DSA, PET, and SPECT, which need to be verified by a surgeon (6). In addition, numerous studies have been conducted on glioblastoma biomarkers, such as the specificity of circRNAs in glioblastoma, which is expected to be a new biomarker for the development of glioblastoma (13) and to lead to a future cure for glioblastoma tumors. In this study, information from clinical sample data was used to study some of the genes that may be correlated with glioblastoma prognosis. Genes significantly associated with glioblastoma prognosis can be identified through a whole-gene batch survival analysis of clinical samples, and then key genes can be further identified by PPI and LASSO. Then, an expression validation analysis of these genes can be performed to identify potential prognostic biomarkers associated with glioblastoma. Finally, independent prognostic factors for glioblastoma can be obtained via an independent prognostic analysis of these genes.

In this study, we used the clinical data of glioblastoma patients available from the TCGA and identified genes associated with glioblastoma prognosis by performing a whole-gene bulk survival analysis. We also investigated the functional enrichment pathways of these genes and found that they were significantly enriched in functions such as building free 40S subunits as well as in placental development. A network of gene–gene interactions was also constructed, and the highest scoring motif modules were further analyzed with the help of algorithms. Eight genes—RPS10, RPS11, RPS19, RSL24D1, RPL39L, EIF3E, NUDT5, and RPF—were found to have an expression in the prognosis of glioblastoma. Finally, univariate cox and multivariate cox analyses were performed, which identified RPL39L and NUDT5 as independent prognostic factors for glioblastoma. The results were verified by qRT-PCR experiments.

Ribosomal proteins are synthesized in the cytoplasm by RNA polymerase II and then imported into the nucleus, where they are assembled into small and large ribosomal subunits (14, 15). The small ribosomal subunit contains an 18S rRNA and approximately 32 ribosomal proteins (RPS proteins), and the large ribosomal subunit 60S consists of one of 5S, 5.8S and 28S rRNAs and approximately 47 ribosomal proteins (RPL proteins) (16). Long-term studies have shown that ribosomal proteins not only constitute ribosomes as structural proteins but also play important roles in the cell cycle, proliferation, apoptosis/death, tumorigenesis, DNA repair, and other responses (17).

From the analysis conducted in this study, it is clear that most of the genes associated with glioblastoma prognosis are ribosomal protein genes, represented by RPS10 and RPS11. The RPS10 gene encodes the RPS10 protein, which is part of the small subunit of the mitochondrial ribosome (18) and is involved in ribosome biogenesis, as well as participating in cellular transformation mechanisms (16). RPS10 encodes the 165 amino-acid-long RPS10 protein, which is a component of the 40S ribosomal subunit (19) and can cross-link to the eukaryotic initiation factor 3 (eIF3) of translation. It has been shown that the RPS10 protein is part of the structural domain involved in the binding of the initiation factor to the 40S subunit at the onset of translation (20). RPS11 encodes the RPS11 protein, which is overexpressed in various malignancies and is associated with tumor recurrence (17). Elevated levels of RPS11 have been found to be associated with a poor prognosis in patients with glioblastoma (21). RPL19 is upregulated in many prostate cancers, and its downregulation leads to a milder malignant phenotype *in vivo*, suggesting a functional role in promoting tumorigenesis (22). It has been shown that RPL19 is associated with glioblastoma prognosis (23). nudt5 encodes NUDT5 hydrolase, which is associated with breast cancer prognosis (24). It can inhibit the propagation of HeLa cells and T47D cells (25). No studies have been conducted on NUDT5 related to glioblastoma.

GO analysis of important pathways has identified the composition of the free 40s subunit pool and placental development, among others, and studies have shown that 40S subunits can enter the free and membrane-bound polyribosomes from the cytoplasmic pool of newly made free natural subunits (26), thus affecting protein synthesis and cell development. There is a link between the placenta and cancer cells at the molecular level (27), and the placenta acts as a transport function between the mother and the fetus, during development. Genes influence tumorigenesis by affecting the 40s subunit and placental development, among others.

In conclusion, this study obtained two independent prognostic markers associated with glioblastoma prognosis, which could play a central role in the prognosis of glioblastoma, were obtained by bioinformatics using glioblastoma tumor samples and normal samples.

## Data availability statement

The original contributions presented in the study are included in the article/supplementary material. Further inquiries can be directed to the corresponding author.

## Author contributions

YT: Conceptualization, Methodology, Writing - Original Draft, Writing - Review & Editing. LC: Conceptualization, Formal analysis, Writing - Original Draft, Writing - Review & Editing. All authors contributed to the article and approved the submitted version.
